# Editorial: The heterogeneity of neuropsychiatric disorders

**DOI:** 10.3389/fpsyt.2022.1114164

**Published:** 2023-01-10

**Authors:** Zhao-Min Wu, Bin-Rang Yang

**Affiliations:** Children's Care and Mental Health Center, Shenzhen Children's Hospital, Shenzhen, China

**Keywords:** heterogeneity, attention-deficit/hyperactivity disorder, obsessive-compulsive disorder, schizophrenia, autism spectrum disorder

## Introduction

Neuropsychiatric disorders, such as attention-deficit/hyperactivity disorder (ADHD), autism spectrum disorder (ASD), obsessive-compulsive disorder (OCD), and schizophrenia (SCZ), are highly heterogeneous. The heterogeneity in psychiatric disorders has still not been fully investigated (1). The current topic, therefore, aims to further illustrate the heterogeneity and explore potential strategies to reduce heterogeneity in neuropsychiatric disorders.

### Heterogeneity within the same diagnostic category

Some of the patients within the same diagnostic category, though not all of them, show some personality traits that might affect their clinical presentation and prognosis (2, 3). For instance, some patients with bipolar disorder (BD) show elevated impulsivity. Zakowicz et al. assessed impulsivity levels in BD patients who had attempted suicide and those who had not. They found impulsivity to be a weak predictor of suicidal risk.

### Overlapping symptoms and cognitive profile

Most neuropsychiatric disorders have overlapping symptoms. Cui et al. used the Child Behavior Checklist (CBCL) to access the clinical profiles of children and adolescents with Tourette syndrome (TS) and compared them to individuals with MDD, ADHD, and OCD. Their results established that TS has a similar emotional and clinical profile to MDD but not ADHD or OCD.

In another example with BD, it is defined as having two phases, depression and hypomania, so its symptoms overlap with those of unipolar depression (UD) during the depression phases. Some patients with depression show soft bipolar signs or bipolarity, e.g., a family history of BD or hyperthymic personality (4). Lu et al. investigated the neuropsychological characteristics of individuals with BD, UD, and depression disorder with bipolarity (UDB). They found that the cognitive dysfunction pattern in patients with UDB is different from that of individuals with UD but similar to that of individuals with BD. Likewise, the aberrant perceptual experience and impaired social communication in ASD and SCZ are probably due to their shared impairment in audiovisual temporal integration (5).

### Shared and distinct neural and genetic correlates

Overlapping symptoms in different diagnostic categories are assumed to have shared underlying mechanisms. The obsessive symptoms in OCD and delusion in SCZ have some overlapping features, such as intrusive and unwanted thoughts. Zhang Y et al. explored the neural correlates of SCZ and OCD using resting-state brain functional imaging techniques. They detected brain activity abnormalities in the right hippocampus and the left posterior cingulate cortex in both SCZ and OCD groups. Liu et al. performed a meta-analysis and found decreased gray matter volume (GMV) in children and adolescents with SCZ and increased GMV in the prefrontal cortex (PFC) in those with OCD. SCZ and MDD also have overlapping symptoms. Ma, Zhang, Zhang, Yan et al. found impaired processing speed and reduced gray matter volume in the medial superior frontal cortex were shared by SCZ and MDD.

In addition to brain morphology and function, genetic characterization of psychiatric disorders has also been shown to transcend diagnostic boundaries. For instance, OCD is often comorbid with other psychiatric disorders, and previous studies have found significant genetic correlations among OCD, MDD (*r* = 0.21) (6), and ADHD (*r* = −0.17) (7). Strom et al. explored the genetic correlations with other somatic and mental illnesses and genetic correlates of OCD in the context of comorbid MDD, ASD, or ADHD. The authors applied multiple approaches to publicly available genome-wide association studies summary statistics and unpublished imputed genotyping data to estimate the genetic relationships among multiple phenotypes robustly. The genetic correlations among the comorbid groups and other somatic and mental illnesses differed from their relationships with OCD-only group and these correlations were affected by comorbidities.

### Diverse etiological factors

Although patients with the same diagnosis often share some core symptoms, they can differ in many ways, e.g., different genetic backgrounds or childhood adverse events. Ma, Zhang, Zhang, Su et al. explored the effects of childhood maltreatment on brain function in patients with major depressive disorder (MDD). Zhang H et al. investigated how the DRD4 −521 C/T SNP affects local brain activity and functional connectivity (FC) in children with ADHD. Both studies identified abnormal brain activation and/or FC that was affected by either environmental factors (e.g., childhood maltreatment) or genetic polymorphism. These results indicated that different etiological factors might be involved in developing psychiatric disorders in different individuals.

### Exploration of novel nosology

Traditional nosology systems define disorders as distinct phenotypes. ADHD is conceptualized three different ways in the Diagnostic and Statistical Manual (DSM-5), the International Classification of Diseases-10 (ICD-10), and the Hierarchical Taxonomy of Psychopathology (HiTOP). To further elucidate the latent structure of ADHD symptoms, Gomez et al. used confirmatory factor analysis (CFA), the exploratory structure equation model (ESEM), and bi-factor S-1 (“asymmetrical”) models in parent and teacher rating scales. Their findings showed that the optimum structure of ADHD symptoms contains only the inattention-specific factor and the g-factor (reflecting impulsivity), consistent with the HiTOP conceptualization of ADHD. Barron et al. proposed in their conceptual analysis article that digital technology, which quantifies human behaviors, may benefit psychiatry in clinical settings.

## Conclusions

The current work explores the multilevel heterogeneity of various psychiatric disorders. These results support the Research Domain Criteria (RDoC) and HiTOP frameworks, which might help promote the classification of psychiatric phenotypes and accelerate progress in studies of psychiatric disorders. More studies based on RDoC and HiTOP would be valuable. “Bottom-up” pathophysiology is of great importance in creating novel nosology and in guiding treatment decisions.

## Author contributions

Z-MW and B-RY developed the article concept. Z-MW wrote the draft of the manuscript. B-RY provided revision suggestions. All authors contributed to the article and approved the submitted version.

## References

[1] Ross CA Margolis RL. Research domain criteria: strengths, weaknesses, and potential alternatives for future psychiatric research. Mol Neuropsychiatry. (2019) 5:218–36. 10.1159/000501797 31768375 PMC6873013

[2] Swann AC. Mechanisms of impulsivity in bipolar disorder and related illness. Epidemiol Psichiatr Soc. (2010) 19:120–30. 10.1017/S1121189X00000828 20815296

[3] Ramirez-Martin A Ramos-Martin J Mayoral-Cleries F Moreno-Kustner B Guzman-Parra J. Impulsivity, decision-making and risk-taking behaviour in bipolar disorder: a systematic review and meta-analysis. Psychol Med. (2020) 50:2141–53. 10.1017/S0033291720003086 32878660

[4] Perugi G Akiskal HS. The soft bipolar spectrum redefined: focus on the cyclothymic, anxious-sensitive, impulse-dyscontrol, and binge-eating connection in bipolar II and related conditions. Psychiatr Clin North Am. (2002) 25:713–37. 10.1016/S0193-953X(02)00023-0 12462857

[5] Zhou H Cui X Yang B Shi L Luo X Cheung EFC. Audiovisual temporal processing in children and adolescents with schizophrenia and children and adolescents with autism: evidence from simultaneity-judgment tasks and eye-tracking data. Clin Psychol Sci. (2022) 10:482–398. 10.1177/21677026211031543

[6] Cross-Disorder Group of the Psychiatric Genomics Consortium. Genomic relationships, novel loci, and pleiotropic mechanisms across eight psychiatric disorders. Cell. (2019) 179:1469–82.e11. 10.1016/j.cell.2019.11.020 31835028 PMC7077032

[7] Strom NI Grove J Meier SM Baekvad-Hansen M Becker Nissen J Damm Als T. Polygenic heterogeneity across obsessive-compulsive disorder subgroups defined by a comorbid diagnosis. Front Genet. (2021) 12:711624. 10.3389/fgene.2021.711624 34531895 PMC8438210

